# Barriers to hand hygiene in ophthalmic outpatients in Uganda: a mixed methods approach

**DOI:** 10.1186/s12348-016-0077-0

**Published:** 2016-03-17

**Authors:** Rachel Mearkle, Rebecca Houghton, Dan Bwonya, Robert Lindfield

**Affiliations:** International Centre for Eye Health, London School of Hygiene and Tropical Medicine, Keppel Street, London, WC1E 7HT UK; Department of Infection, University Hospital Southampton NHS Foundation Trust, Tremona Road, Southampton, SO16 6YD UKᅟ; Eye Unit, Mengo Hospital, Plot 396, Albert Cook Road, PO Box 7161, Kampala, Uganda

## Abstract

**Background:**

Healthcare-associated infection rates are high in low-income countries and are associated with significant morbidity. There is a paucity of published data on infection control practice, attitudes or resources in these settings, particularly in ophthalmology. The aim of this study is to understand current hand washing practices, barriers to hand washing and facilities available in two Ugandan specialist eye hospitals. This study was undertaken through non-participant observations of healthcare worker hand washing practices, documentation of hand hygiene facilities and semi-strucutured interviews with clinical staff.

**Results:**

Eighty percent of the WHO opportunities for hand washing were missed through lack of attempted hand hygiene measures. Facilities for hand hygiene were inadequate with some key clinical areas having no provisions for hand hygiene. Training on effective hand hygiene varied widely with some staff reporting no training at all. The staff did not perceive the lack of facilities to be a barrier to hand washing but reported forgetfulness, lack of time and a belief that they could predict when transmission might occur and therefore did not wash hands as often as recommended.

**Conclusions:**

Hand hygiene at the two observed sites did not comply with WHO-recommended standards. The lack of facilities, variable training and staff perceptions were observable barriers to effective hand hygiene. Simple, low-cost interventions to improve hand hygiene could include increased provision of hand towels and running water and improved staff education to challenge their views and perceived barriers to hand hygiene.

## Background

Healthcare-associated infection (HCAI) is a major global problem with an estimated prevalence of 15.5 episodes per 100 patients in developing countries [1]. In comparison, European prevalence rates have been estimated to be 5.7 % [2]. Significant contamination of clinician’s hands and medical equipment occurs after each patient interaction [3, 4], and hand hygiene is the main barrier against transmission of potential pathogens [5]. HCAIs carry a high economic burden in developing countries, and this is also where simple interventions such as effective hand hygiene may have the greatest impact [6].

There is a significant burden of HCAI in Africa [7], but the understanding of infection control practice has been limited to small single-centre studies [8–13]. The importance of infection control in ophthalmology has been highlighted in high-income settings [3, 14], but we identified only one published audit of infection control practices in developing world ophthalmology [11]. Whilst there are reports of nosocomial-acquired infection in ophthalmology centres [15–18] and despite the potentially devastating outcomes including visual loss and permanent disability, there is very little data on the role of infection prevention and control.

We aimed to evaluate current hand hygiene practice and identify any true or perceived barriers to effective hand hygiene using mixed methods. Triangulation of methodologies, using both qualitative and quantitative approaches, enabled a greater understanding of practice, facilities and staff attitudes to effective hand hygiene and any existing barriers to optimal practice recommended by the WHO.

## Methods

Observations of hand hygiene, inventory of facilities and interviews with staff were conducted in two eye units in Uganda in February 2014 for 1 week at each hospital consecutively. For this study, hospitals were randomly allocated as hospital A and hospital B. Interviewees were given a random identifying number to maintain confidentiality. This study was conducted as part of a larger study evaluating the role of mentorship in the quality of cataract surgery; for which ethical approval was provided by the London School of Hygiene and Tropical Medicine, Mbarara University of Science and Technology, the Medical Research Council of Uganda and both hospitals.

### Observations

Observations were performed using the WHO Patient Safety Observation Form [19] to document healthcare workers hand hygiene actions, based on the WHO five moments (indications) for hand hygiene (see Table 1). Data was collected on ‘opportunities’ for hand hygiene which occurred before or after one or more ‘indications’ for hand hygiene. Indications were as follows: before patient care, before aseptic procedure, after contact with body fluids, after patient contact and after contact with patient surroundings. During the observation period, opportunities, indications and hand hygiene actions were recorded. Hand hygiene actions were hand washing or use of alcohol hand rub. The quality of the hand hygiene action was not recorded. Common indications for a hand hygiene opportunity in this setting included touching the patient, administering eye drops and slit lamp examination of patients. Observations were carried out by two researchers (RM, DB), medical doctors trained in the UK and Uganda, respectively. The observation tool was piloted prior to use and discussed with the research team. Simple descriptive statistics were performed in Excel using chi-squared test to assess differences between the hospitals.

**Table 1:** Summary of details collected on the WHO observation form. The form is designed to aid in the collection of data when observing the hand hygiene activity of HCWs in different clinical settings. It has three main categories

Category	Details collected
Identifying details	A range of details are recorded about the location of the observation (e.g. the department and ward), the timing (day, time at the start and end of observation period) and the initials of the observer.
Person being observed	There is a column to identify each individual being observed and record their professional category (e.g. nurse or doctor).
Opportunities for hand hygiene	Each row details the opportunities for hand hygiene for each individual being observed. This includes the indication (e.g. before touching a patient or after touching a patient’s surroundings) as well as details of any resultant hand hygiene action (if gloves or hand rub were used, if hands were washed or if an opportunity was missed).

WHO Observation Form available from URL: http://www.who.int/gpsc/5may/Observation_Form.doc?ua=1

### Inventory

An inventory of hand hygiene resources was conducted in each hospital on two different days at different times. The data collection tool was adapted from a previously used resource [12] and is available on request. Data was captured on facilities for hand hygiene in each clinical area and the convenience and cleanliness of these facilities. The data was collected by two researchers (RM, DB) who concurrently observed the facilities and recorded the results. Descriptive statistical analysis was performed in Microsoft Excel.

### Interviews

Clinical staff were recruited for interviews through convenience sampling until saturation was reached. A review of existing literature and discussion with experts led to the development of an interview framework which included a range of questions exploring views of hand hygiene and potential barriers to hand hygiene. Questions were open ended with prompts if required. The interview framework was piloted to explore clarity and comprehension of the questions (see Table 2). The structure and purpose of the interviews were discussed with each member of the staff before the interview and they gave written consent to take part in the study including their interviews being digitally recorded. All interviews were conducted in English.

**Table 2:** Hand hygiene interview framework

Theme	Example questions
Training	Have you been trained on how to wash your hands?
	If yes—when was the training? How long was the training? What did you learn on the training? Who provided the training?
Importance	Do you think that hand washing is important? Why/Why not?
	If you do not wash your hands, does it cause any problems?
	Why do you wash your hands at all?
Knowledge	How should you wash your hands?
	How often should you wash them?
Hospital facilities	Can you wash your hands as often as you would like in this hospital? If no—why not?
	Do you have everything you need to wash your hands here? If no—what is missing?
Barriers	What stops you washing your hands more often?
	What would make it easier for you to wash your hands?
	What could the hospital do to make it easier for you to wash your hands more effectively/frequently?

All interviews were conducted in a private room by a single interviewer (RL). The interviews were recorded digitally and subsequently transcribed verbatim. A thematic analysis was undertaken using an inductive approach to identify themes. Coding was an iterative process; codes initially related to the interview framework, but after reviewing, the interview codes were revised. Themes, codes and sub-codes were reviewed by RH and RM before a final version of the coding system was developed. Themes were identified at a semantic level. A definition was applied to each code and a code book was developed and used for refining the coding process (code dictionary available on request). Nvivo 10 was used to organise the codes and aid analysis. Thematic categories were developed after codes were finalised.

## Results

### Observations

Ten observations were carried out in each site, ranging from 10 to 32 min, with an average of 13.4 min; 268 min of observation was recorded in total. Fifty-six different patient interactions were observed with 37 staff members (although some staff members were observed on more than one occasion). Clinical areas observed included clinical room on the ward, clinical outpatients and visual acuity testing in two different eye units. The staff observed included nurses, ophthalmologists, clinical officers and student nurses.

In total, 288 opportunities for hand hygiene were observed and 57 hand hygiene actions were taken; overall, 80 % of hand hygiene opportunities during the observation period were missed. The percentage of missed opportunities were similar in the two hospitals; 79 % of opportunities were missed in hospital A, compared with 82 % in hospital B (*p* = 0.5).

**Fig. 1:**
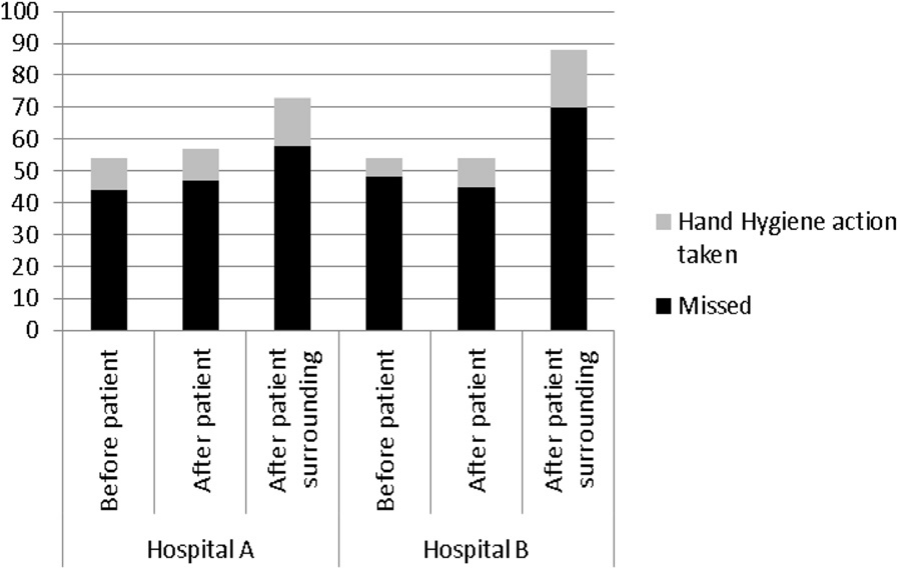
Number of opportunities missed and where hand hygiene action was taken by hospital and indication

Figure 1 shows that the most common indication for hand hygiene action observed was after a health professional touched a patient’s surroundings. This accounted 42 % of indications observed and was the indication most likely to result in a hand hygiene action being taken. Overall, the staff were most likely to miss the indication of ‘before touching a patient’.

**Fig. 2:**
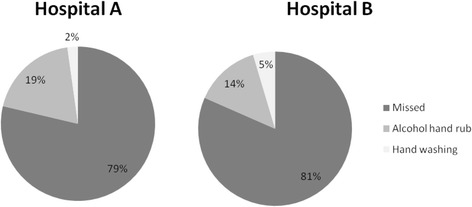
Outcome of hand hygiene opportunities by hospital

Gloves were rarely worn, being observed only four times in total, and only at one hospital (hospital B). Figure 2 shows that hand hygiene with alcohol-based rub accounted for the vast majority of hand hygiene actions observed: 47 out of 57 observed hand hygiene (81 %). Alcohol-based rubs were more frequently used in hospital A; accounting for 90 % of hand hygiene actions in hospital A, compared with 75 % of hand hygiene actions taken in hospital B.

### Inventory

An inventory of hand hygiene facilities was conducted over both sites in multiple clinical areas on two separate occasions by two researchers RM and DB. In total, data was collected in 4 wards, 3 inpatient clinical rooms and 15 outpatient rooms.

See Table 3 for a summary of the findings. Hospital B had a lack of adequately functioning sinks; there were four nonfunctioning sinks observed on the ward or in outpatients. Only 25 % of outpatient rooms had a functioning sink with soap and adequate hand-drying facilities on both observations. There were similar findings on the ward, where neither male or female bays nor the treatment room had any working provision for hand hygiene.

**Table 3:** Inventory of hand hygiene facilities

	Hospital A		Hospital B	
	Inpatient	Outpatient	Inpatient	Outpatient
Percentage of clinical areas^a^ with fully functioning sinks^b^ on both observations	33 %	100 %	25 %	25 %
Percentage of clinical areas^a^ with adequate hand-drying facilities^c^ on both observations	0 %	0 %	25 %	25 %
Percentage of clinical areas^a^ with alcohol hand rub on both observations	66 %	91 %	25 %	100 %

^a^Clinical areas in inpatient mean female bay, male bay, examination room and treatment room. Clinical areas in outpatients are examination and treatment rooms

^b^Fully functioning sinks mean running cold tap, sink which is connected to plumbing and soap available

^c^Adequate hand-drying facilities mean multiple cloth hand towels and bin for used hand towels

In hospital A, all of the clinical outpatient rooms had functioning sinks as did the ward areas (but some of those lacked soap); however, hand-drying facilities were inadequate. Hand towels were sometimes provided but there was often just one cloth towel and no receptacle for used towels, which is likely to lead to reuse of hand towels. In hospital B, there were 4.57 clean cloth hand towels on average per sink; but in hospital A, there were just 0.75 clean cloth towels on average.

In both hospitals, there were areas where lack of facilities might have impacted on clinical care. Hospital B had three clinical areas (one inpatient treatment room, a male bay and a female bay) with no useable hand hygiene facilities at all. In hospital A, all sinks were functioning; however, one clinical area (the male bay) had no soap at the sink and no alcohol rub available. In both hospitals, alcohol hand rub was widely available in outpatient clinical areas.

1. Clinical areas in inpatient mean female bay, male bay, examination room and treatment room. Clinical areas in outpatients are examination and treatment rooms.

### Interviews

Nine interviews were conducted as part of a longer interview with staff which was completed at the end of a 1-year study on the impact of a mentor on quality of cataract surgery. HCWs included theatre nurses, outpatient nurses, clinical officers and ophthalmologists.

Two people reported that they had never received any hand hygiene training, and six people reported that they had only received training as an undergraduate. Only two people (one at each hospital) mentioned that the hospital had offered training in the last year. The training the people had received was variable; and although most described that the training provided a process of hand washing, they struggled to recall this. For example, two HCWs mentioned a five-stage washing technique although neither could recall what the five stages were. Only one person described that they were taught that they should wash between each patient.

Most staff (5 of 9) reported that they washed hands between each patient.

'[I clean] every after patient because we have got bottles of alcohol on every slit lamp there is a bottle of alcohol so every after patient you have got to spray and it dries.' (A3)

Of the other staff, one reported that they wash their hands ‘each time [they] handle anything’ (B3), and three others reported less frequent washing; ‘after at least, like after 3–4 patients I need to wash my hands’ (B4).

All staff reported that they had adequate facilities in the hospital to perform hand hygiene, and no one identified inadequate facilities as a barrier to hand washing. Staff felt that hand hygiene was important, and they showed a good understanding of transmission of infection through direct contact.

'[Hand hygiene is] important, you can spread infection you can get that from another patient and you take it, and you can also spread it to another patient.' (A4)

Staff reported that the primary aim of hand hygiene was to prevent spread between patients, but some staff also identified transmission to healthcare workers and surroundings as important.

Although the staff demonstrated an understanding about the importance of hand washing, there was a widespread belief that one could assess who was infectious and change your hand hygiene behaviour to provide protection, with some conflicting views on the relative effectiveness of alcohol.

‘I use alcohol whenever I see that that patient is very infectious.’ (A2)

'Especially if it’s an infective case then I usually do not use only alcohol that’s when I go for the soap and water.' (B2)

When barriers to hand washing were discussed, the staff highlighted that a busy ‘workload’ (B5) and forgetfulness were issues that could be a barrier to hand washing.

'Sometimes you find that you have seen three or four patients and you don’t [clean your hands].... you are picking bacteria and transferring it, my bacteria may not be yours… We know it but somehow we don’t. I think its carelessness..... Alcohol is easier to pick but sometimes you even forget about it.' (A1)

The staff found that the convenience of alcohol rubs enables more regular hand cleaning, although two members of staff identified its smell as a barrier at hospital A; but this was not reported at hospital B.

'The type of alcohol we are using, some of us feel intoxicated somehow because it has got a strong [fumes]… to others I think that could be a barrier.' (A3)

## Discussion

This study showed that hand hygiene was not conducted as frequently as recommended by the WHO guidance [20]. The main reasons for this included limited facilities for hand hygiene and staff perceptions that hand hygiene, whilst important, was not necessarily required for all patients. The interviews also identified that there is a belief that just some patients are identifiably ‘infectious’ and that hand hygiene behaviour could be altered as a result. Whilst all staff reported that facilities for hand hygiene were adequate, the observations and inventory identified that staff were not cleaning their hands as often as recommended and that there was a critical lack of facilities.

Previous studies have shown that HCWs do not wash their hands as often as is recommended; a systematic review in high-income countries reported a rate of compliance with guidelines of 40 % [21]. Few studies of infection control practice have been published from Africa [8–13], and we only identified one focusing on ophthalmology. This study reported a bacteriological survey of post-surgical eye infections in Nigeria; like this study, it also identified a lack of hand-drying facilities as an issue [11]. Other studies support the findings of this study; that lack of facilities for hand hygiene made regular hand cleaning difficult [13] and that lack of training on hand cleaning may also play a role [10].

Studies have shown that being observed (the ‘Hawthorn effect’) can impact hand hygiene behaviours [22, 23]; therefore, this study may have overestimated the number of hand hygiene actions performed in each hospital. However, in this case, it would not have been possible for the research team to make observations without being noticed. In addition, the WHO observation tool was restricted in what it collected, so data was not available regarding the distribution of gloves or the quality of the hand hygiene action [19]. Due to the limitations on time, there were insufficient numbers of observations to reliably identify differences between professional groups about their hand hygiene behaviours. Despite these weaknesses, the study presents results from two very different eye units: presenting a range of different professional behaviours and views, and it does this in a multifaceted way with qualitative and quantitative data to allow a nuanced understanding of the hand hygiene culture in the hospital.

## Conclusions

This study has highlighted issues with the facilities for hand hygiene as well as deficiencies in hand hygiene behaviours and beliefs of staff. Future training and education in this setting need to challenge these beliefs and practices in order to improve the frequency of hand hygiene. The WHO reports that promotion of hand hygiene is cost effective [20], and even simple measures like improving access to alcohol hand rub have been shown to be effective [24]. In resource-poor environments, the prioritisation of hand hygiene is vital so that scarce resources are allocated appropriately as part of the patient safety agenda.
